# Digitalization for supply chain resilience and robustness: The roles of collaboration and formal contracts

**DOI:** 10.1007/s42524-022-0229-x

**Published:** 2023-02-08

**Authors:** Ying Li, Dakun Li, Yuyang Liu, Yongyi Shou

**Affiliations:** 1 School of Management, Shandong University, Jinan, 250100 China; 2 School of Management, Zhejiang University, Hangzhou, 310058 China

**Keywords:** digitalization, supply chain, resilience, obustness, collaboration, formal contract

## Abstract

Black swan events such as the coronavirus (COVID-19) outbreak cause substantial supply chain disruption risks to modern companies. In today’s turbulent and complex business environment, supply chain resilience and robustness as two critical capabilities for firms to cope with disruptions have won substantial attention from both the academia and industry. Accordingly, this study intends to explore how digitalization helps build supply chain resilience and robustness. Adopting organizational information processing theory, it proposes the mediating effect of supply chain collaboration and the moderating effect of formal contracts. Using survey data of Chinese manufacturing firms, the study applied structural equation modelling to test the research model. Results show that digitalization has a direct effect on supply chain resilience, and supply chain collaboration can directly facilitate both resilience and robustness. Our study also indicates a complementary mediating effect of supply chain collaboration on the relationship between digitalization and supply chain resilience and an indirect-only mediation effect on the relationship between digitalization and supply chain robustness. Findings reveal the differential roles of digitalization as a technical factor and supply chain collaboration as an organizational factor in managing supply chain disruptions. Paradoxically, formal contracts enhance the relationship between digitalization and supply chain resilience but weaken the relationship between supply chain collaboration and supply chain resilience. The validation of moderating effects determines the boundary conditions of digitalization and supply chain collaboration and provides insights into governing supply chain partners’ behavior. Overall, this study enhances the understanding on how to build a resilient and robust supply chain.

This work was supported by the National Natural Science Foundation of China (Grant Nos. 71902102 and 72010107002).

## 1 Introduction

The coronavirus (COVID-19) outbreak causes substantial supply chain (SC) disruption risks to modern companies (Ivanov, 2020; El Baz and Ruel, 2021). Accenture (2022) reported that 94% of Fortune 1000 companies are seeing SC disruptions from the COVID-19 pandemic. The highly turbulent and complex environment drives the firm to enhance SC resilience and robustness to deal with disruption risks, which are recognized as two critical capabilities in the risk management literature (Brandon-Jones et al., 2014; El Baz and Ruel, 2021; Ruel and El Baz, 2021). SC resilience is the ability to proactively prepare for and rapidly respond to disruptive events (Bag et al., 2021; Bahrami and Shokouhyar, 2022), and SC robustness reflects the ability to maintain the continuity of operations and the stability of the SC system (Brandon-Jones et al., 2014; Durach et al., 2015). The positive effects of SC resilience and robustness on firm's performance have won widespread recognition, both at the firm level (Ruel and El Baz, 2021; Bahrami and Shokouhyar, 2022) and the SC level (Juan et al., 2022). However, a comprehensive answer remains lacking on how firms can build SC resilience and robustness, which is particularly urgent due to black swan events such as the COVID-19 pandemic (Ivanov and Dolgui, 2020).

In the context of Industry 4.0, practitioners and scholars have extensively discussed digitalization in the field of SC management. Digitalization is the adoption of digital technologies to create new processes or transform existing processes to adapt to the changing business environment (Eller et al., 2020; Song et al., 2021; Zhou et al., 2021).

The development and adoption of emerging digital technologies are speeding up the manufacturing firm's digitalizing process, which has been steadily affecting themselves and their SC activities (Li et al., 2020). Current research has examined the impact of individual digital technologies on SC resilience, such as blockchain (Dubey et al., 2020), artificial intelligence (Belhadi et al., 2021), and big data analytics (Bag et al., 2021; Dubey et al., 2021; Bahrami and Shokouhyar, 2022; Iftikhar et al., 2022). However, few studies have investigated the impact of digitalization on SC robustness. Given the difference between SC resilience and robustness (Brandon-Jones et al., 2014; El Baz and Ruel, 2021; Ruel and El Baz, 2021), the impact of digitalization on SC resilience and robustness may differ but remains unexamined.

Organizational information processing theory (OIPT) is adopted as the theoretical lens of this study to investigate how digitalization can contribute to SC resilience and robustness (Galbraith, 1974; Premkumar et al., 2005). OIPT emphasizes the match between information-processing requirements and information-processing capabilities (Galbraith, 1974; Premkumar et al., 2005). SC risks are caused by uncertainties along the SC, and SC risk management activities are highly information-intensive (Fan et al., 2017). Digitalization could help enhance the firm's information-processing capability in reacting to SC disruptions and maintaining SC stability (Premkumar et al., 2005; Fan et al., 2017; Dubey et al., 2020; 2021). However, digitalization alone may be inadequate for such tasks in the SC context (Shou et al., 2021). Digitalization provides the digital infrastructure for collaboration among SC partners, which further promotes companies to share risk information and respond quickly to changes in the environment (Scholten and Schilder, 2015) and hence contributes to the establishment of SC resilience and robustness (Juan et al., 2022). Therefore, based on OIPT, we posit that digitalization (as a technical factor) can improve SC resilience and robustness through SC collaboration (as an organizational factor).

We also intend to investigate how formal contracts moderate the impact of digitalization and SC collaboration on SC resilience and robustness. Digitalization has not only transformed the original way of SC operations but also left some hidden dangers (e.g., opportunism, information leakage, information disruption, and data tampering) (Ivanov et al., 2019; Yang et al., 2021; Son et al., 2021; Kessler et al., 2022). Meanwhile, firms struggle to seek ways of managing and governing collaboration with SC partners due to capability asymmetry and opportunism (Um and Oh, 2020). Regarding the exchange hazards related to digitalization and SC collaboration, scholars have advocated using formal contracts to coordinate and control SC partners' information exchange and processing behaviors (Son et al., 2021; Kessler et al., 2022). Transaction cost economics suggests that formal contracts can regulate exchange activities and mitigate exchange hazards by specifying the roles and responsibilities of each SC partner in legally predefined terms (Cao and Lumineau, 2015; Um and Oh, 2020). However, few studies have empirically investigated the influence of formal contracts on the relationships among digitalization, SC collaboration, SC resilience, and SC robustness.

Against this backdrop, this study attempts to explore impacts of digitalization on SC resilience and robustness and proposes the mediating role of SC collaboration and the moderating role of formal contracts. Using a survey of the Chinese manufacturing industry, we test the hypothesized relationships. This study contributes to the relevant literature in three aspects. First, we construct the links between digitalization and two dimensions of the ability to manage SC disruption risks (i.e., resilience and robustness), which deepen the understanding of the role of digitalization in the turbulent business environment. Second, we validate the mediating effect of SC collaboration, revealing the underlying mechanisms of how digitalization leads to resilience and robustness and pointing out the path to build a resilient and robust SC. Third, formal contracts are introduced as a moderating variable, which determines the boundary condition of digitalization and SC collaboration. Moreover, our study provides managerial insights on how to improve SC resilience and robustness through digitalization, collaboration, and proper arrangements of formal contracts to deal with today's turbulent business environment.

## 2 Theoretical background and hypothesis development

### 2.1 Organizational information processing theory

Modern organizations are often confronted with complex tasks that require them to organize, process, and utilize information efficiently (Srinivasan and Swink, 2018; Dubey et al., 2021). OIPT emphasizes the "fit" between information-processing capabilities and requirements to ensure organizational performance (Galbraith, 1973; 1974). Based on OIPT, uncertainty or complexity caused by the lack or inefficient use of information brings significant information-processing needs and requires high information-processing capabilities (Galbraith, 1973; 1974). Thus, the organization is suggested to decrease information-processing needs by creating slack resources and self-contained tasks or improve information-processing capabilities by designing organizational structures and mechanisms, and applying technologies (Galbraith, 1973; 1974; Premkumar et al., 2005).

The efforts to build SC resilience and robustness are highly information-intensive (Fan et al., 2017). SC partners need to collect and exploit the information for timely and accurate decision making to tackle disruptive events (Belhadi et al., 2021; El Baz and Ruel, 2021). Hence, based on OIPT, this study argues that to enhance SC resilience and robustness, the focal firm in the SC has to improve information-processing capabilities (Wong et al., 2020). Digitalization and SC collaboration could be considered as practices to enhance information-processing capabilities (Li et al., 2020; Dubey et al., 2021). However, the underlying mechanisms are different. Digitalization facilitates information collection and analysis through technological approaches (Li et al., 2020; Dubey et al., 2021), which is in accordance with the OIPT strategies of "investment in vertical information systems" (Galbraith, 1974). SC collaboration promotes information exchange, mutual understanding, and joint decision making through management approaches (Cao and Zhang, 2011), which is fairly consistent with the "creation of lateral relations" and "long-term associations or coalitions" proposed by Galbraith (1974). Digitalization establishes the digital infrastructure for SC collaboration to exchange and integrate high-quality information from SC partners, which further help deal with risks and improve resilience and robustness.

Thus, based on OIPT, we propose that digitalization and SC collaboration improve SC resilience and robustness. Moreover, formal contracts in our study regulate the SC partners' information-processing behaviors through formal rules, procedures, and programs (Cao and Lumineau, 2015; Um and Oh, 2020), and could further influence the effectiveness of digitalization and SC collaboration. We therefore suggest that formal contracts moderate the impact of digitalization and SC collaboration on SC resilience/robustness. Figure 1 presents the conceptual model of our study.

## 2.2 Impact of digitalization on SC resilience and robustness

According to the OIPT, digitalization can improve the organization's information-processing capability (Li et al., 2020) and contribute to building SC resilience capability in the pre- and post-disruption aspects. First, digitalization can improve the organization's capability to prepare for SC-related emergencies by gathering internal production and external SC information (Li et al., 2020). The companies can apply digital technologies (e.g., big data analytics) to monitor their SC operations in real time, identify the sources of SC disruption risks, determine the root causes of problems and defects, and thus develop effective risk prevention plans (Wang et al., 2016; El Baz and Ruel, 2021). Second, when a disruptive event occurs, digitalization can enable the companies to collect a wealth of information from various sources throughout the SC to support quick decision making and timely risk response (Bahrami and Shokouhyar, 2022). With the abundant information available, SC managers can clearly understand disruptions and the corresponding causes and quickly propose response strategies (Wang et al., 2016). Third, prior experience, information, and knowledge of dealing with disruptive events contribute to the improvement of SC resilience (Ambulkar et al., 2015). Digital technologies can facilitate the digitization and standardization of historical experience, knowledge, and information from inside and outside the company and enable the storage and retrieval of digitized information in the databases (Xue et al., 2013). Based on historical information, the organization can handle disruptive events more efficiently (Bag et al., 2021; Belhadi et al., 2021). Therefore, the following hypothesis is proposed:

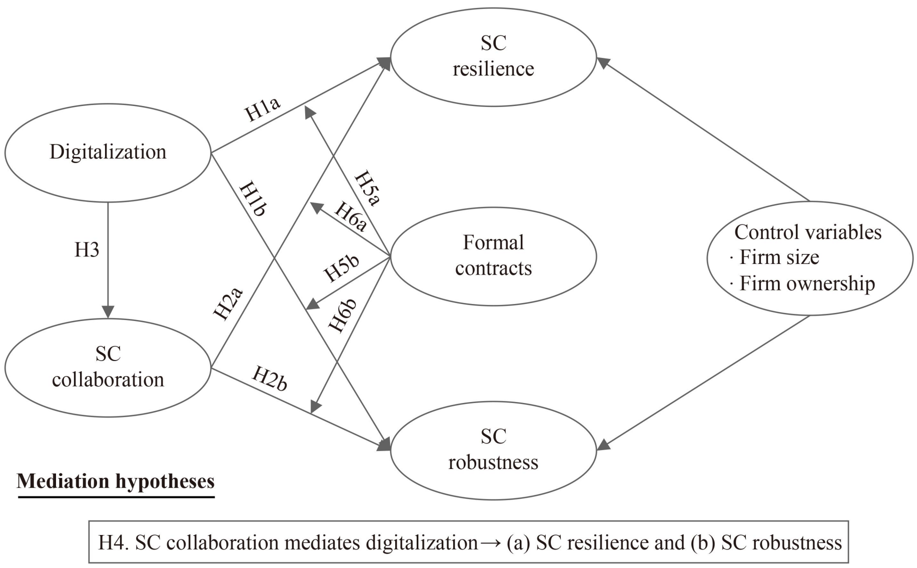

**Fig. 1** Conceptual model.

**H1a.** Digitalization has a positive impact on SC resilience.

SC robustness indicates the organization's ability to maintain a stable SC system (Durach et al., 2015). Brandon-Jones et al. (2014) pointed out that a robust SC system often requires flexible changes to accommodate the specific disturbance. Digitalization can flexibly reconfigure the digitized resources distributed in the SC system, enhance the risk absorption capacity, and thus ensure its robustness (Ivanov et al., 2019). Digital technology can help obtain timely information about customers' changing demands and suppliers' fluctuant inventory levels, improving end-to-end visibility and flexible response to changes in demand and supply (Williams et al., 2013; Wang et al., 2016; Srinivasan and Swink, 2018). Additionally, scholars propose that building redundancy (e.g., in reserves or back-up options) is a commonly used measure to improve SC robustness (Durach et al., 2015). The companies can efficiently set up and manage slack resources based on the improved information-processing capabilities through digitalization. For example, Roßmann et al. (2018) indicated that big data analytics can be used to manage safety stocks which help absorb the adverse impact of SC risks. Companies can also adopt digital technologies to create a data-driven learning system that simulates disruption scenarios on the basis of historical information to generate various possible solutions (Ivanov and Dolgui, 2021). In a word, digitalization ensures SC robustness by efficiently managing slack resources and developing back-up solutions to maintain the continuity of SC operations without weakening SC performance. Therefore, we propose the following hypothesis:

**H1b.** Digitalization has a positive impact on SC robustness.

### 2.3 Impact of SC collaboration on SC resilience and robustness

The companies can collaborate with SC partners to proactively make contingency plans and quickly identify and respond to disruptive risks (Bag et al., 2021; Bahrami and Shokouhyar, 2022), thereby improving SC resilience. First, SC collaboration enhances the willingness of SC partners to share high quality demand and supply information, which could improve the visibility and transparency of the SC system (Srinivasan and Swink, 2018; Dubey et al., 2020). Multi-channel and frequent information exchange among SC partners guarantees comprehensive and timely information related to potential disruptive risks for the companies. Notably, they serve as the preconditions for accurate risk identification and proper risk planning. Second, SC collaboration enables quick and efficient decision making by engaging in joint efforts and aligning goals with SC partners (Cao and Zhang, 2011; Scholten and Schilder, 2015). Consequently, the SC exhibits resilience by quickly and accurately responding to disruptions in a volatile environment. Remarkably, analyzing disruptive events is a necessary but possibly cost-ineffective and complex task, especially for small companies. Joint actions and common goals with SC partners can help the company deal with complex risk information and make consistent and rational decisions to address disruption risks (Scholten and Schilder, 2015). On the basis of the above analysis, we propose the following hypothesis:

**H2a.** SC collaboration has a positive impact on SC resilience.

This study proposes that a collaborative SC relationship can help the companies build a robust SC structure to resist external disturbances. Bensaou and Venkatraman (1995) indicated that collaboration with SC partners could enable the firm to meet the customers' demand continuously. IKEA, for example, builds a strong collaborative relationship with SC partners and provides timely information of real-time changes in customer demand to its long-term suppliers. IKEA's suppliers can therefore promptly respond to market changes and anticipate and avoid disruptive risks in advance, representing a high level of SC robustness. Moreover, SC partners tend to make flexible and dynamic adjustments while attaining common goals (Cao and Zhang, 2011; Scholten and Schilder, 2015). When disruptions may threaten the operation of SC system, the companies can be motivated to work together with suppliers and customers with common goals to maintain the original SC performance. SC partners involved in a collaborative relationship behave as a coherent unity which can react to disruptions and fulfill the predefined goals by harnessing collective intelligence (Barratt, 2004; Scholten and Schilder, 2015). Therefore, the SC operations would be able to continue, and performance would not reflect substantial deviations. The following hypothesis is proposed:

**H2b.** SC collaboration has a positive impact on SC robustness.

### 2.4 Mediating effect of SC collaboration

This study argues that digitalization provides a digital infrastructure for collaboration among SC partners. Li et al. (2020) advocated that the adoption of digital technologies facilities the building of a digital SC platform which enables companies to better exchange and transfer information with their partners. Based on rich demand and supply information collected through digital technologies, companies can select proper SC partners to quickly build a strong supply network and establish well-organized SC relationships (Nayal et al., 2022). Moreover, digitalization improves information transparency throughout the whole SC (Aben et al., 2021; Dubey et al., 2021). Adequate information can help enhance mutual understanding and trust among SC partners, which are basic elements of SC collaboration (Dubey et al., 2020). Digitalization can also help standardize and integrate SC processes (Zouari et al., 2021), lower the transaction costs among SC partners, and ensure interoperability between heterogeneous organizations (Nayal et al., 2022). In this case, digitalization encourages collaborative behaviors among SC members. Hence, we propose that

**H3.** Digitalization has a positive impact on SC collaboration.

Our study further proposes that SC collaboration mediates the effect of digitalization on SC resilience and robustness. Digitalization provides an information exchange channel for SC partners by adopting digital technologies (Li et al., 2020), while SC collaboration further ensures the sources of accurate and timely information by establishing lateral relationships (Michalski et al., 2018). Without mutual trust and understanding, SC partners can scarcely connect with each other to share critical information (Michalski et al., 2018; Um and Oh, 2020). SC collaboration can also promote the integration of internal and external information collected through digital technologies (Li et al., 2020). The information-sharing behaviors enabled by SC collaboration and information flow channels provided by digital technologies break down the information silos in the SC system, help truly deliver end-to-end information, and further enhance the focal firm's information-processing capabilities (Dubey et al., 2020; Gebhardt et al., 2022). In this case, the high-quality information flows across the whole supply chain can help better identify and deal with potential SC risks, thus improving resilience and robustness.

Moreover, digitalization allows the extensive collection of information, providing SC partners with the basis for joint decision making to ensure resilience and robustness. Digital technology adoption increases the quantity of available information and helps alleviate information uncertainty (Roßmann et al., 2018). In this case, the companies can easily identify potential disruptive events. However, the vast amount of information may also bewilder managers and prevent the extraction of valuable insights (Williams et al., 2013). Based on the tremendous amount of information collected through digitalization (Ivanov et al., 2019), SC partners can extract valuable information and create useful knowledge through joint decision making (Puranam et al., 2006; Belhadi et al., 2021) to deal with SC risks. Scholars also emphasized that digitalization can be used effectively to deal with SC risks that occur frequently and regularly (Keller et al., 2021). In the context of unexpected SC disruptions, digitalization shows a big limitation of intelligent decision making and creates a need for SC collaboration to make joint efforts (Scholten and Schilder, 2015). Based on the above analysis, we propose the following hypothesis:

**H4.** SC collaboration mediates the impact of digitalization on (a) SC resilience and (b) SC robustness.

### 2.5 Moderating effect of formal contracts

Our study argues that formal contracts can strengthen the effects of digitalization on SC resilience and robustness by improving information quality and security. Indeed, the implementation of digitalization improves the ability to exchange and share information along the SC (Zouari et al., 2021) but may also induce some potential behavioral risks (Yang et al., 2021; Kessler et al., 2022). Formal contracts serve as a control and coordination mechanism to minimize idiosyncratic and deviant behaviors and enable SC partners to attain desired outcomes (Poppo and Zenger, 2002). When implementing digitalization, the companies can craft formal contracts to clearly define the rights and responsibilities of SC partners (Poppo and Zenger, 2002). The contract can encourage the SC partners to establish a coordinated and prescribed exchange and processing of information through digital technologies. Moreover, formal contracts often contain provisions of penalties for non-compliance and violation behaviors (Lee and Cavusgil, 2006). In such circumstances, opportunism risks related to information exchange and processing can be mitigated, which can help ensure information integrity and credibility (Cao and Lumineau, 2015; Aben et al., 2021). In this case, formal contracts complement digital technologies to ensure resilience and robustness (Keller et al., 2021; Son et al., 2021). Therefore, we propose the following hypothesis:

**H5.** Formal contracts positively moderate the impact of digitalization on (a) SC resilience and (b) SC robustness.

We speculate that formal contracts may weaken the effect of SC collaboration on SC resilience and robustness. SC collaboration promotes SC resilience and robustness through the relational mechanisms of trust and joint decision making. Malhotra and Murnighan (2002) indicated that contractually binding SC partners' behavior may sow the seeds of mutual suspicion. When engaging in a collaborative relationship, SC partners pursue mutual trust and work together to achieve common goals to build resilience and robustness (Cao and Zhang, 2011). Um and Oh (2020) pointed out that formal contracts legally and strictly specify the roles, responsibilities, and penalties to the detriment of trust in a collaborative SC relationship. Contracts may harm the sense of trust and intimacy between SC partners, thus affecting the efficiency of SC operations and damaging the joint efforts to cope with disruption risks (Cao and Lumineau, 2015).

In addition, the drafting, development, monitoring, and regular adjustment of complex contracts consume numerous resources and costs from all SC partners (Poppo and Zenger, 2002; Son et al., 2021), largely restricting the flexibility of SC collaboration and undermining the level of resource readiness to manage disruptions (Cao and Lumineau, 2015). All formal contracts are inevitably incomplete (Poppo and Zenger, 2002). When unforeseen disruptions occur, SC partners involved in formal contracts can scarcely rely on appropriate clauses and may create new conflicts and disputes. Even if procedures for addressing sudden risks are specified in the contracts, SC partners need to go through pre-arranged mechanisms accordingly (Keller et al., 2021). The predefined processes in the formal contracts would reduce the flexibility of information exchange and processing between SC partners, leading to ineffective decision making and premature risk response strategies (Um and Oh, 2020; Keller et al., 2021). In summary, we propose the following hypothesis:

**H6.** Formal contracts negatively moderate the impact of SC collaboration on (a) SC resilience and (b) SC robustness.

## 3 Method

### 3.1 Sampling and data collection

We applied a survey-based method to test the proposed hypotheses in the Chinese manufacturing sector. We first designed a reliable and valid questionnaire by reviewing the relevant literature. The back-translation method was utilized to ensure consistency across languages in the instruments. Moreover, we requested three senior researchers and nine purchasing and supply chain managers to evaluate the structure, comprehensibility, and completeness of the questionnaire to ensure content validity. A pilot study of 50 managers was then conducted to determine the feasibility of the study protocol and validate the measurement instruments.

To maximize the response rate, this study used a convenience sampling method to collect data. Salganik and Heckathorn (2004) indicated that convenience samples can also produce asymptotically unbiased estimates. The potential respondents included middle- and high-level managers owing to their good understanding of applications of new technologies and SC management activities. Following the guideline of Dillman (2011), we distributed the online questionnaire to potential respondents from manufacturing firms located in the key economic zones of China, including the Circum-Bohai-Sea region, the Pan-Pearl River Delta, and the Yangtze River Delta. Finally, we distributed 1681 questionnaires and received 290 completed valid responses, with a 17.3% response rate. We tested non-response bias by comparing the differences in firm size and firm ownership between early and late respondents. The *t*-test showed no significant difference, indicating that non-response bias is not an issue in our study. Table 1 summarizes the demographic statistics of the sampled firms.

**Table 1** Sample profile

| | Freq. | Percentage (%) |
|---|---|---|
| **Industry** | | |
| Publishing and printing | 3 | 1.03 |
| Electronic and electrical equipment | 74 | 25.52 |
| Textile and apparel | 12 | 4.14 |
| Arts and crafts | 3 | 1.03 |
| Chemicals and petrochemicals | 14 | 4.83 |
| Building materials | 14 | 4.83 |
| Metal, mechanical, and engineering | 59 | 20.35 |
| Wood and furniture | 5 | 1.72 |
| Food beverage and alcohol | 18 | 6.21 |
| Toys | 3 | 1.03 |
| Rubber and plastics | 11 | 3.79 |
| Pharmaceutical and medical | 14 | 4.83 |
| Equipment | 58 | 20.00 |
| Jewelry | 2 | 0.69 |
| **Firm sales (million yuan)** | | |
| $\leqslant$ 20 | 70 | 24.14 |
| 21–50 | 40 | 13.79 |
| 51–100 | 60 | 20.69 |
| 101–400 | 69 | 23.79 |
| > 400 | 51 | 17.59 |
| **Number of employees** | | |
| $\leqslant$ 50 | 9 | 3.10 |
| 51–100 | 34 | 11.73 |
| 101–300 | 66 | 22.76 |
| 301–500 | 55 | 18.97 |
| 501–1000 | 61 | 21.03 |
| 1001–2000 | 17 | 5.86 |
| > 2000 | 48 | 16.55 |

Following the suggestions of Podsakoff and Organ (1986) and Podsakoff et al. (2003), we addressed the potential common method bias through ex ante and post ante measures. First, the independent and dependent constructs were separated in the questionnaire to avoid the double-barreled problem. Second, items in the questionnaire were adapted from existing literature and pretested by experts to avoid ambiguity. In addition, 27.9% of the respondents were in top management positions (e.g., president, CEO, and director), while 72.1% were from the middle management level (e.g., manager of SC, IT, marketing, purchasing, and production). These knowledgeable respondents can accurately understand and answer the survey questions. Finally, the results of Harman's single factor analysis indicate that the first of the four extracted factors explained only 32% of the total variance. Therefore, common method bias was unlikely to be an issue in our research.

### 3.2 Measures

SC resilience and robustness are two critical capabilities to deal with SC disruption risks (El Baz and Ruel, 2021). SC resilience is defined as the capability to proactively prepare for and rapidly respond to SC disruptions (Bag et al., 2021; Bahrami and Shokouhyar, 2022). The measures include five items (Jia et al., 2020; Belhadi et al., 2021). SC robustness refers to the ability to maintain stable operations and planned performance (El Baz and Ruel, 2021; Ruel and El Baz, 2021). We apply four items to measure SC robustness (Brandon-Jones et al., 2014).

Digitalization refers to the adoption of digital technologies to transform intra- and inter-organization activities (Eller et al., 2020; Yang et al., 2021; Zhou et al., 2021). The measures of digitalization include five items based on prior studies (Eller et al., 2020; Son et al., 2021; Song et al., 2021; Yang et al., 2021; Zhou et al., 2021). SC collaboration is defined as sharing information, resources, and knowledge to achieve mutual objectives together with SC partners (Cao and Zhang, 2011). SC collaboration was measured by five items (Cao and Zhang, 2011; Dubey et al., 2020; Um and Oh, 2020; Gebhardt et al., 2022; Juan et al., 2022).

Formal contracts are regarded as a moderator in our research model. Cao and Lumineau (2015) described formal contracts as the adoption of the written and detailed contract to safeguard inter-organizational relationships and reduce opportunism and conflicts. Accordingly, five items were used to measure formal contracts.

All the above measures were summarized in Table 2 and examined by five-point Likert scales in which 1

**Table 2** Reliability and validity

| Constructs and items | | Factor loadings | SD | T-statistics |
|---|---|---|---|---|
| Digitalization (DT) (Cronbach's $\alpha = 0.753$; CR = 0.835; AVE = 0.503) | | | | |
| DT1 | Our firm adopts digital technologies to collect data from different sources | 0.687 | 0.048 | 14.278 |
| DT2 | Our firm adopts digital technologies to connect business processes | 0.668 | 0.043 | 15.623 |
| DT3 | Our firm adopts digital technologies to promote information exchange | 0.684 | 0.045 | 15.268 |
| DT4 | Our firm adopts digital technologies to build customer interface | 0.747 | 0.033 | 22.678 |
| DT5 | Our firm adopts digital technologies to connect supply chain partners | 0.756 | 0.035 | 21.752 |
| Supply chain collaboration (SCC) (Cronbach's $\alpha = 0.753$; CR = 0.835; AVE = 0.503) | | | | |
| SCC1 | Our firm sets common goals with supply chain partners | 0.706 | 0.041 | 17.288 |
| SCC2 | Our firm shows mutual support with supply chain partners | 0.681 | 0.044 | 15.640 |
| SCC3 | Our firm engages in joint problem solving with supply chain partners | 0.697 | 0.033 | 21.260 |
| SCC4 | Our firm shows understanding of supply chain partners' strengths and weaknesses | 0.695 | 0.037 | 18.865 |
| SCC5 | Our firm engages in regular and collaborative communication with supply chain partners | 0.765 | 0.032 | 23.705 |
| Supply chain resilience (SCRES) (Cronbach's $\alpha = 0.805$; CR = 0.865; AVE = 0.561) | | | | |
| SCRES1 | Our firm can predict supply chain disruption risk occurrence | 0.759 | 0.029 | 26.166 |
| SCRES2 | Our firm develops contingency plans to prepare for potential supply chain disruptions | 0.752 | 0.033 | 22.974 |
| SCRES3 | Our firm can prevent disruptions through forecasting and planning | 0.717 | 0.035 | 20.684 |
| SCRES4 | Our firm can adapt to supply chain disruptions by quickly re-engineering the processes | 0.761 | 0.029 | 26.033 |
| SCRES5 | Our firm can respond quickly to SC disruptions | 0.754 | 0.031 | 24.276 |
| Supply chain robustness (SCROB) (Cronbach's $\alpha = 0.674$; CR = 0.799; AVE = 0.501) | | | | |
| SCROB1 | Operations would be able to continue | 0.812 | 0.027 | 30.467 |
| SCROB2 | We would still be able to meet customer demand | 0.696 | 0.038 | 18.136 |
| SCROB3 | Our firm can avoid losses caused by supply chain risks | 0.633 | 0.053 | 11.857 |
| SCROB4 | Performance would not deviate significantly | 0.677 | 0.055 | 12.393 |
| Formal contracts (FC) (Cronbach's $\alpha = 0.767$; CR = 0.843; AVE = 0.519) | | | | |
| FC1 | Our firm has specific and detailed contractual agreements with supply chain partners | 0.742 | 0.037 | 19.967 |
| FC2 | The contract specifies and details the roles and responsibilities of supply chain partners | 0.745 | 0.033 | 22.540 |
| FC3 | The contract specifies and details how to deal with unexpected events | 0.689 | 0.045 | 15.286 |
| FC4 | The contract specifies and details the penalties for under-performance | 0.781 | 0.028 | 28.181 |
| FC5 | The contract specifies and details how to deal with disputes and conflicts | 0.638 | 0.043 | 14.750 |

Note: SD, CR, and AVE are short for standard deviation, composite reliability, and average variance extracted, respectively.

indicates “strongly disagree” and 5 indicates “strongly agree”. In addition, firm size measured by the number of employees was introduced as a control variable (Brandon-Jones et al., 2014; Belhadi et al., 2021; Juan et al., 2022). Large firms have rich resources to conduct SC risk management practices (Ruel and El Baz, 2021; El Baz and Ruel, 2021). We also controlled for firm ownership because state-owned enterprises (SOEs) in China have advantages in gaining resources and support from the government in case of disruptions (White, 2000; Cuervo-Cazurra et al., 2014) and hence may show better resilience and robustness than privately owned enterprises.

### 3.3 Data analysis technique

Partial least squares structural equation modeling (PLS-SEM) is a variance-based statistical model (Hair et al., 2016) and is widely employed to test causal relationships among multiple constructs (Hair et al., 2016; 2019). Many studies have applied PLS-SEM in the research field of SC management (Peng and Lai, 2012; Sturm et al., 2022). The advantages of PLS-SEM summarized by Hair et al. (2019) match well with our research. First, PLS can analyze complex models to provide acceptable results without requiring a large sample size (Peng and Lai, 2012; Michalski et al., 2018). Kock and Hadaya (2018) suggested that the minimum required sample size for PLS-SEM is 146 based on gamma-exponential method or 160 based on inverse square root method. The sample size of this study is 290, which meets the requirement. Second, PLS-SEM does not require the data to strictly follow the assumption of normal distribution (Hair et al., 2016), which happens to be suitable for a small sample size. Small samples tend to have a non-normal distribution, and in this case, the results employing PLS-SEM are more reliable than covariance-based SEM (Reinartz et al., 2009). Third, the prediction-oriented PLS-SEM is suitable for exploratory studies to derive theory development and extension (Hair et al., 2019). The current study considers digitalization a new antecedent to SC resilience and robustness. It introduces SC collaboration as a mediating variable and formal contracts as a moderating variable. We aim to better explain how digitalization enhances SC resilience and robustness and expand OIPT with the integration of technological and organizational factors.

## 4 Analyses and results

### 4.1 Measurement model assessment

The standardized root mean square residual (SRMR) and the root mean squared residual covariance matrix of the outer model residuals (RMS_theta) were employed to evaluate the overall quality of a reflective measurement model. A value of SRMR less than 0.08 and RMS_theta below 0.12 indicates a good model fit (Henseler et al., 2016). The results in our study show that SRMR being 0.062 and RMS_theta being 0.119 satisfy the requirement.

We then assessed (i) indicator reliability and internal consistency reliability, (ii) convergent validity, and (iii) discriminant validity of the measurement model. Our study can establish indicator reliability, as all factor loadings to their corresponding constructs were above the 0.6 threshold with $t$-value being larger than 2 (Comrey, 1973; Anderson and Gerbing, 1988). Cronbach’s $\alpha$ values were above the limit of 0.6 (Nunnally, 1978). Composite reliability (CR) values of all the constructs were greater than 0.7 as shown in Table 2, exceeding the minimum value of 0.6 (Hair et al., 2019). The average variance extracted (AVE) values were greater than the cut-off score of 0.5, which demonstrates the convergent validity (Hair et al., 2019). Then, we applied three criteria to check discriminant validity. First, for all constructs, the square roots of AVE of the constructs were higher than each of their possible pairwise correlation in Table 3 (Fornell and Larcker, 1981). Second, the heterotrait-monotrait ratio (HTMT) values were within the threshold of 0.9 (Hair et al., 2016; 2019). Third, the factor loadings were all higher than the corresponding cross-loadings as presented in Table 4. Thus, these results established discriminant validity.

### 4.2 Structural model analysis

Figure 2 presents the established structural model to test hypothesized relationships. The quality of our structural model was first assessed. We calculated the variance inflation factor (VIF) to examine collinearity. All VIF values were less than 3.0, suggesting that multi-collinearity was not an issue in our study (Hair et al., 2019). Then, the coefficient of determination ($R^2$) and the cross-validated redundancy measure ($Q^2$) were calculated to indicate

**Table 3** Correlations, square root of the AVE values, and the HTMT values

| Constructs | DT | SCC | SCRES | SCROB | FC |
|---|---|---|---|---|---|
| DT | **0.709** | | | | |
| SCC | 0.428<br>(0.559) | **0.709** | | | |
| SCRES | 0.484<br>(0.617) | 0.575<br>(0.726) | **0.749** | | |
| SCROB | 0.316<br>(0.431) | 0.542<br>(0.721) | 0.651<br>(0.876) | **0.708** | |
| FC | 0.314<br>(0.411) | 0.639<br>(0.835) | 0.639<br>(0.806) | 0.483<br>(0.643) | **0.721** |

Note: The square root of AVE is on the diagonal in bold, and the HTMT values are in parentheses.

**Table 4** Cross loadings

| Items | DT | SCC | SCRES | SCROB | FC |
|---|---|---|---|---|---|
| DT1 | **0.687** | 0.287 | 0.327 | 0.193 | 0.208 |
| DT2 | **0.668** | 0.264 | 0.319 | 0.200 | 0.199 |
| DT3 | **0.684** | 0.252 | 0.353 | 0.201 | 0.202 |
| DT4 | **0.747** | 0.385 | 0.347 | 0.269 | 0.246 |
| DT5 | **0.756** | 0.313 | 0.371 | 0.249 | 0.252 |
| SCC1 | 0.303 | **0.706** | 0.358 | 0.401 | 0.356 |
| SCC2 | 0.277 | **0.681** | 0.337 | 0.328 | 0.420 |
| SCC3 | 0.361 | **0.697** | 0.431 | 0.398 | 0.432 |
| SCC4 | 0.258 | **0.695** | 0.419 | 0.384 | 0.500 |
| SCC5 | 0.310 | **0.765** | 0.476 | 0.405 | 0.547 |
| SCRES1 | 0.376 | 0.480 | **0.759** | 0.484 | 0.499 |
| SCRES2 | 0.396 | 0.438 | **0.752** | 0.447 | 0.517 |
| SCRES3 | 0.349 | 0.427 | **0.717** | 0.495 | 0.467 |
| SCRES4 | 0.375 | 0.446 | **0.761** | 0.509 | 0.476 |
| SCRES5 | 0.305 | 0.344 | **0.754** | 0.510 | 0.419 |
| SCROB1 | 0.276 | 0.521 | 0.527 | **0.812** | 0.430 |
| SCROB2 | 0.211 | 0.380 | 0.469 | **0.696** | 0.401 |
| SCROB3 | 0.197 | 0.293 | 0.387 | **0.633** | 0.223 |
| SCROB4 | 0.197 | 0.275 | 0.448 | **0.677** | 0.258 |
| FC1 | 0.223 | 0.516 | 0.496 | 0.358 | **0.742** |
| FC2 | 0.273 | 0.459 | 0.473 | 0.320 | **0.745** |
| FC3 | 0.216 | 0.464 | 0.397 | 0.334 | **0.689** |
| FC4 | 0.239 | 0.494 | 0.505 | 0.374 | **0.781** |
| FC5 | 0.177 | 0.360 | 0.419 | 0.350 | **0.638** |

Note: Bold values are loadings for items on the corresponding construct, which are all above the threshold value of 0.6.

the explanatory power and predictive accuracy of the structural model, respectively (Hair et al., 2016; 2019).

Based on the results shown in Table 5, the structural model can explain 18.3% of SC collaboration, 40.1% of SC resilience, and 31.0% of SC robustness. The $Q^2$ values were larger than zero. The above results demonstrate that our research model has a strong explanatory and predictive power.

Table 6 summarizes the structural model analysis results with 5000 bootstrapping resamples. H1a was supported, with a positive relationship between digitalization and SC resilience ($\beta = 0.288$, $p < 0.001$). However, we found no significant direct impact of digitalization on SC robustness ($\beta = 0.084$, $p > 0.05$). Hence, H1b was rejected. According to the results, SC collaboration significantly impacted SC resilience ($\beta = 0.450$, $p < 0.001$) and SC robustness ($\beta = 0.496$, $p < 0.001$), supporting both H2a and H2b. The effects of firm size and firm ownership were controlled for, and the corresponding results were insignificant in this study. H3 hypothesized that digitalization positively influences SC collaboration and was supported with $\beta$ being 0.428 and $p$ value being 0.

We tested the mediation effects of SC collaboration following the guidelines of Zhao et al. (2010) and Nitzl et al. (2016). Table 7 shows that digitalization has positive and significant indirect effects on both SC robustness and SC resilience via SC collaboration, given that the indirect path coefficients for DT→SCC→SCRES and DT→SCC→SCROB being 0.193 and 0.212 were all significant. As suggested by Zhao et al. (2010), the significance of the indirect path demonstrates the mediation, while the direct effect determines the type of mediation. Considering that the mediated effect and direct effect of digitalization on SC resilience both existed and pointed at the same directions, a complementary mediation effect of SC collaboration on the relationship between digitalization and SC resilience was validated (Zhao et al.,

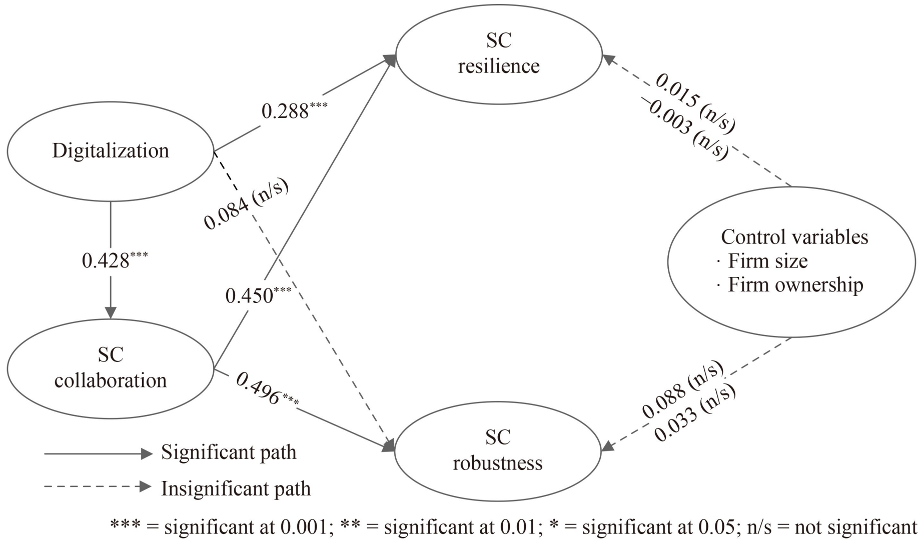

**Fig. 2** Structural model results for direct effects.

**Table 5** Quality of the structural model

| Constructs | $R^2$ | $Q^2$ |
|---|---|---|
| DT | – | – |
| SCC | 0.183 | 0.084 |
| SCRES | 0.401 | 0.206 |
| SCROB | 0.310 | 0.134 |

2010). However, H1b was not supported, indicating no direct effect of digitalization on SC robustness. Therefore, an indirect-only mediation effect of SC collaboration was confirmed (Zhao et al., 2010). In short, both H4a and H4b were supported.

Furthermore, we tested the moderation effects of formal contracts by adding the interaction terms into the structural model as shown in Fig. 3. The interaction terms were built on the basis of the two-stage calculation method with standardized indicators (Henseler and Chin, 2010). Table 8 presents the results. H5a and H5b hypothesized that formal contracts positively moderate the impact of digitalization on SC resilience and SC robustness, respectively. H5a was supported because the interaction term of digitalization and formal contracts showed a significant and positive impact on SC resilience ($\beta$ = 0.129, 0.01 < $p$ < 0.05). However, the influence of the interaction term on SC robustness was insignificant ($\beta$ = 0.092, $p$ > 0.05), and the direct effect of digitalization on SC robustness was also not demonstrated, thereby not supporting H5b. Based on a similar logic, the moderation effect of formal contracts on the relationship between SC collaboration and SC resilience was demonstrated to be significantly negative ($\beta$ = −0.104, 0.01 < $p$ < 0.05). Thus, H6a was supported. However, the moderating effect of formal contracts on the relationship between SC collaboration and SC robustness was insignificant ($\beta$ = −0.079, $p$ > 0.05). Therefore, H6b was not supported.

## 5 Discussion

### 5.1 Findings

This study first empirically examines the impact of digitalization on SC resilience and robustness. Digitalization is found to be positively related to SC resilience. However, no empirical evidence is found to support the significant direct impact of digitalization on SC robustness. When faced with SC disruptions caused by black swan events such as the COVID-19 outbreak, manufacturing firms can scarcely maintain stable production and SC operations by relying on digitalization alone. The lack of resources may also cause SC disruptions and thus weaken firm performance. For example, in the automotive industry, the chip shortage crisis which occurred in 2020 has directly led to a decline in vehicle production and an extension of lead time. Although the degree of SC digitalization in the automobile industry is relatively high, the supply–demand imbalance of chips remains unsolved and negatively affects SC robustness.

The mediating effect of SC collaboration is validated. The results demonstrate a complementary mediation effect of SC collaboration on the relationship between digitalization and SC resilience. It means that digitalization can partially lead to SC resilience through SC collaboration and implies an unexplained direct effect of digitalization on SC resilience (Zhao et al., 2010). Moreover, this study reveals an indirect-only mediation effect of SC collaboration on the relationship between

**Table 6** Direct effect testing results

| Paths | Coefficient ($\beta$) | SD | T-statistics | $P$-values | Inference |
|---|---|---|---|---|---|
| H1a. DT→SCRES | 0.288*** | 0.059 | 4.864 | 0.000 | Supported |
| H1b. DT→SCROB | 0.084 | 0.056 | 1.493 | 0.135 | Not supported |
| H2a. SCC→SCRES | 0.450*** | 0.053 | 8.552 | 0.000 | Supported |
| H2b. SCC→SCROB | 0.496*** | 0.052 | 9.620 | 0.000 | Supported |
| H3. DT→SCC | 0.428*** | 0.054 | 7.988 | 0.000 | Supported |
| Firm ownership→SCRES | −0.003 | 0.046 | 0.070 | 0.945 | – |
| Firm ownership→SCROB | 0.033 | 0.055 | 0.605 | 0.545 | – |
| Firm size→SCRES | 0.015 | 0.053 | 0.278 | 0.781 | – |
| Firm size→SCROB | 0.088 | 0.060 | 1.473 | 0.141 | – |

**Table 7** Mediating effect testing results

| Specific indirect paths | Coefficient ($\beta$) | SD | T-statistics | $P$-values | Inference |
|---|---|---|---|---|---|
| H4a. DT→SCC→SCRES | 0.193 | 0.034 | 5.624 | 0.000 | Supported |
| H4b. DT→SCC→SCROB | 0.212 | 0.036 | 5.869 | 0.000 | Supported |

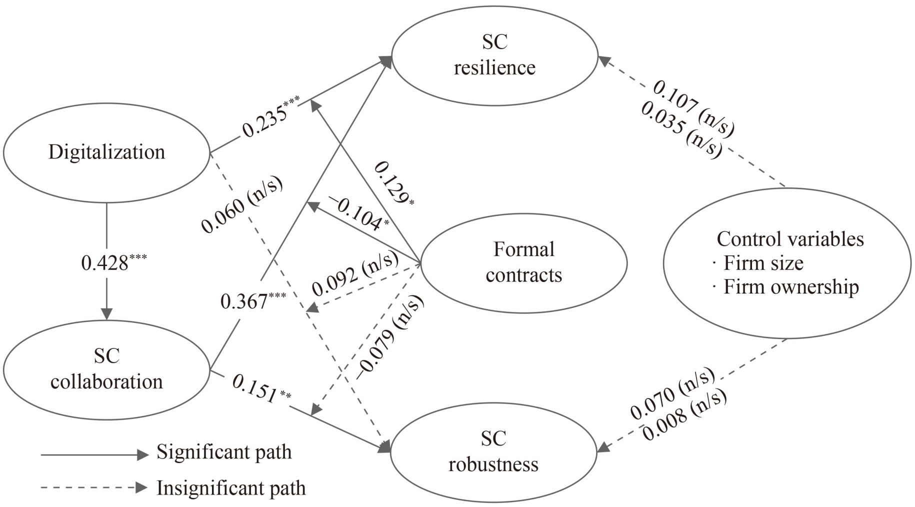

*** = significant at 0.001; ** = significant at 0.01; * = significant at 0.05; n/s = not significant

**Fig. 3** Structural model results for moderating effects.

**Table 8** Moderating effect testing results

| Moderating effect paths | Coefficient ($\beta$) | SD | T-statistics | *P*-values | Inference |
|---|---|---|---|---|---|
| H5a. DT*FC→SCRES | 0.129* | 0.062 | 2.067 | 0.039 | Supported |
| H5b. DT*FC→SCROB | 0.092 | 0.048 | 1.913 | 0.056 | Not supported |
| H6a. SCC*FC→SCRES | −0.104* | 0.049 | 2.147 | 0.032 | Supported |
| H6b. SCC*FC→SCROB | −0.079 | 0.055 | 1.430 | 0.153 | Not supported |

digitalization and SC robustness. Although digitalization cannot directly improve SC robustness, a significant indirect effect of digitalization exists on SC robustness through SC collaboration.

Our study introduces formal contracts as a moderating variable in the research model. The results support that formal contracts positively moderate the effect of digitalization on SC resilience. Since digitalization does not directly influence SC robustness, the moderating effect of formal contracts on the relationship between digitalization and SC robustness is not established. Formal contracts are found to weaken the effect of SC collaboration on resilience. The use of formal contracts reduces the flexibility of information exchange and processing, particularly when SC partners face unexpected disruptions (Cao and Lumineau, 2015; Um and Oh, 2020). However, formal contracts do not moderate the effects of SC collaboration on SC robustness. We argue that SC resilience emphasizes the actions or behaviors to identify and respond to disruptions. Given the nature of dependence on behaviors, SC resilience requires the match between formal contracts and collaboration to coordinate and constrain SC partners' joint actions. However, compared with SC resilience, SC robustness highlights the stable status of SC network, operations, and performance, which does not rely excessively on SC partners' real-time interactive behaviors. In this case, the role of formal contracts in governing behaviors is only slightly demonstrated.

## 5.2 Theoretical implications

This study contributes to the relevant literature in three aspects. First, the findings in our study enhance the understanding of the distinct effects of digitalization on SC resilience and robustness. SC resilience and robustness are considered two critical dimensions of the organization's capabilities to manage SC disruptive risks (Brandon-Jones et al., 2014; El Baz and Ruel, 2021; Ruel and El Baz, 2021). Although some studies identify SC robustness as a dimension or a subordinate part of SC resilience (Durach et al., 2015; Juan et al., 2022), Afraz et al. (2021) indicated that differences must exist between the two capabilities, and unclear conceptualization may adversely affect the effectiveness of SC management initiatives. Our study differentiates SC resilience and robustness and indicates that digitalization only has a direct and positive effect on SC resilience. The findings provide empirical evidence for the distinct relationships between digitalization and resilience/robustness and hence enrich the digitalization research and SC disruption research.

Second, the mediating effect of SC collaboration can help better explain how digitalization leads to SC resilience and robustness. Apart from revealing whether digitalization influences SC resilience and robustness, more importantly, our study explains how the phenomenon occurs by introducing SC collaboration as mediator. Previous studies have recognized that inter-organizational practices should evolve with the process of digitalization (Dubey et al., 2020; Li et al., 2020). However, few studies have empirically explored how organizational factors match technology adoption in managing SC disruptions. Our study indicates a complementary mediating effect of SC collaboration on the relationship between digitalization and SC resilience and an indirect-only mediation effect on the relationship between digitalization and SC robustness. The validation of the mediating effects of SC collaboration enhances our understanding of the inherent mechanisms of the links from digitalization to SC resilience and robustness and identifies the path to build a resilient and robust SC.

Third, this study examines the moderating effects of formal contracts, which can help understand the boundary conditions under which digitalization and SC collaboration are effective in resolving disruption risks. Previous studies have pointed out that digitalization may result in data integrity and security issues (Favoretto et al., 2022), data tampering and inter-organizational conflicts (Roßmann et al., 2018), information interruption risk (Ivanov et al., 2019), and information asymmetries (Kessler et al., 2022). Our study supports the research of Xue et al. (2013), Aben et al. (2021), Son et al. (2021), Keller et al. (2021), and Kessler et al. (2022) by confirming that certain governance measures should be adopted to cope with the risks and challenges of digitalization. Furthermore, scholars have proposed formal contracts and relational governance mechanisms to coordinate and safeguard SC partners' behaviors (Lee and Cavusgil, 2006). Others explored the interplay between the two governance mechanisms (Poppo and Zenger, 2002; Cao and Lumineau, 2015). SC collaboration characterized by information sharing, mutual trust and understanding, and joint efforts can be viewed as one of the relational governance mechanisms (Barratt, 2004; Cao and Zhang, 2011). The findings of our study indicate that formal contracts and relational mechanisms function as substitutes in coping with disruptions. The findings provide advanced knowledge on how to govern SC partners in case of disruptions.

### 5.3 Managerial implications

The findings also offer practical implications for manufacturing firms. First, firms are suggested to promote digitalization and SC collaboration to build a resilient and robust SC in the current volatile business environment. The firm can adopt more targeted digital solutions and establish digital infrastructure to support information exchange and sharing with SC partners. For example, in the turbulent fast fashion industry, SHEIN, as a global e-retailor, not only leverages its own digital capabilities to rapidly track market demands, but also develops an intelligence collaboration system for SC partners to establish a timely response to demand variability. Thus, SHEIN and its suppliers maintain competitive advantages through small lot sizes, multiple batches, and fast delivery, thereby effectively reducing the risk of inventory and demand changes and ensuring SC resilience and robustness.

Second, firms should leverage formal contracts to flexibly coordinate and control SC partners' behaviors. On the one hand, the managers can design clear contractual provisions to clarify SC partners' roles and responsibilities in digital transformation, establish penalties for non-compliance and violations to mitigate potential opportunism risks, and provide solutions to disputes and emergencies. In this case, the firm can overcome the challenges such as information security and asymmetries related to digitalization. On the other hand, the contract should protect and encourage collaborative behaviors between SC partners in good faith. Certain settings in the contract such as high frequency reporting requirements or excessive supervision may damage trust and destroy the collaborative relationship. We recommend firms to develop more pragmatic and flexible win–win contracts to ensure that the SC system can be sufficiently adaptive to handling conflicts or crises.

## 6 Conclusions

This study explores the impact of digitalization on SC resilience and robustness and proposes the mediating role of SC collaboration and the moderating role of formal contracts from the perspective of OIPT. A survey in the Chinese manufacturing sector was conducted, and structural equation modeling method was applied to test the hypotheses. The results confirm that digitalization is only directly related to SC resilience, while SC collaboration leads to both SC resilience and robustness. Furthermore, SC collaboration mediates the effects of digitalization on SC resilience and robustness. The empirical results reveal that formal contracts positively moderate the impact of digitalization on SC resilience and negatively moderate the impact of SC collaboration on SC resilience. The findings contribute to the current literature related to digitalization, SC resilience, and SC robustness and offer practical implications for manufacturing firms, especially in the turbulent and sophisticated business environment nowadays.

Despite the contributions of this study, some limitations are inevitable and thus drive the need for future research. First, we find a complementary mediation effect of SC collaboration on the relationship between digitalization and SC resilience and an indirect mediation effect of SC collaboration on the relationship between digitalization and SC robustness. Thus, future research can explore other mediators to comprehensively explain how digitalization leads to SC resilience and robustness. Second, this study applied cross-sectional survey data to test the hypotheses. Future researchers should conduct in-depth longitudinal case studies to understand the development of digitalization and collaborative relationship overtime and the subsequent effects. Third, the data were collected from the Chinese manufacturing sector. Future studies can broaden their scope by collecting data from multiple countries and industries and comparing the potential differences in the hypothesized relationships.

**Open Access** This article is licensed under a Creative Commons Attribution 4.0 International License, which permits use, sharing, adaptation, distribution and reproduction in any medium or format, as long as you give appropriate credit to the original author(s) and the source, provide a link to the Creative Commons licence, and indicate if changes were made.

The images or other third party material in this article are included in the article's Creative Commons licence, unless indicated otherwise in a credit line to the material. If material is not included in the article's Creative Commons licence and your intended use is not permitted by statutory regulation or exceeds the permitted use, you will need to obtain permission directly from the copyright holder. To view a copy of this licence, visit http://creativecommons.org/licenses/by/4.0/.
